# Human Chorionic Gonadotropin modulates CXCL10 Expression through Histone Methylation in human decidua

**DOI:** 10.1038/s41598-020-62593-9

**Published:** 2020-04-01

**Authors:** Michelle Silasi, Yuan You, Samantha Simpson, Janina Kaislasuo, Lubna Pal, Seth Guller, Gang Peng, Rosanna Ramhorst, Esteban Grasso, Shervin Etemad, Sandy Durosier, Paulomi Aldo, Gil Mor

**Affiliations:** 1 Yale University School of Medicine, Department of Obstetrics, Gynecology, and Reproductive Sciences, New Haven, CT USA; 2 C.S. Mott Center for Human Growth and Development, Department of Obstetrics, Gynecology, Wayne State University, Detroit, MI USA; 3 Department of Obstetrics and Gynecology, University of Helsinki and the Helsinki University Hospital, Helsinki, Finland; 4 Department of Biostatistics, School of Public Health, Yale University, New Haven, CT USA; 5 Laboratory of Immunopharmacology, University of Buenos Aires School of Sciences, IQUIBICEN-CONICET (National Research Council), Buenos Aires, Argentina

**Keywords:** Immunology, Molecular medicine

## Abstract

The process of implantation, trophoblast invasion and placentation demand continuous adaptation and modifications between the trophoblast (embryonic) and the decidua (maternal). Within the decidua, the maternal immune system undergoes continued changes, as the pregnancy progress, in terms of the cell population, phenotype and production of immune factors, cytokines and chemokines. Human chorionic gonadotropin (hCG) is one of the earliest hormones produced by the blastocyst and has potent immune modulatory effects, especially in relation to T cells. We hypothesized that trophoblast-derived hCG modulates the immune population present at the maternal fetal interface by modifying the cytokine profile produced by the stromal/decidual cells. Using *in vitro* models from decidual samples we demonstrate that hCG inhibits *CXCL10* expression by inducing H3K27me3 histone methylation, which binds to Region 4 of the *CXCL10* promoter, thereby suppressing its expression. hCG-induced histone methylation is mediated through EZH2, a functional member of the PRC2 complex. Regulation of *CXCL10* expression has a major impact on the capacity of endometrial stromal cells to recruit CD8 cells. We demonstrate the existence of a cross talk between the placenta (hCG) and the decidua (*CXCL10*) in the control of immune cell recruitment. Alterations in this immune regulatory function, such as during infection, will have detrimental effects on the success of the pregnancy.

## Introduction

The coordinated balance between the invading trophoblast and a receptive maternal decidua is critical for the success of pregnancy^1,2^. The process of implantation, trophoblast invasion and placentation demand continuous adaptation and modifications between the trophoblast (embryonic) and the decidua (maternal)^3–5^. Within the decidua, the maternal immune system undergoes continued modifications, as the pregnancy progresses, in terms of the cell population, phenotype and production of immune factors, cytokines and chemokines^6^. These adaptation processes are essential for the normal progression of the pregnancy.

The decidua, the pregnant uterine endometrium, has long been considered as a supportive environment for the immune cells and the trophoblast present at the implantation site. However, growing evidence suggest that decidual stromal cells (DSCs) may play a more active role in the regulation of differentiation, migration and function of uterine immune cells^7^ as well as in the protection against infections^8^.

Immune cells are a major cellular component of the human and rodents’ pregnant uterus, and their specific role has been an area of active research. During the first trimester of the human pregnancy, 70% of the leukocytes found in the decidua are Natural Killer (NK) cells, 20% to 25% are macrophages, and approximately 1.7% are dendritic cells (DCs)^9–11^. In addition, the uterine T cell population expands across gestation, and are mostly regulatory in nature^12^. The recruitment of immune cells into the uterus is cell specific and their function is locally controlled^7^. Examples include the increase of NK cells at the end of the menstrual cycle, or recruitment of macrophages and DCs during the preimplantation period^13^. Human endometrial stromal cells (HESCs) are known to produce and secrete various chemokines, including Interleukin-8 (IL-8)^14,15^, growth-regulated oncogene (GRO) α^16–18^, monocyte chemoattractant protein-1 (MCP-1)^15,19^, macrophage inflammatory protein-1 (MIP-1α)^20^, regulated upon activation normal T cell expressed and secreted (RANTES)^21,22^ and interferon-induced protein 10 (IP-10)^23^. The expression of these cytokines has been suggested to be important in menstruation, infections (bacterial and viral), implantation and in the support of early pregnancy^24,25^. Interestingly, the maintenance of pregnancy requires the inhibition of many of these cytokines/chemokines in order to restrict the recruitment of immune cells, such cytotoxic CD8 T cells that could endanger the acceptance of the fetus. Furthermore, several reports suggest that during pregnancy, the decidua is responsible for actively restricting access of cytotoxic CD8 T cells to the maternal-fetal interface^26^ suggesting that the process of differentiation from endometrial stromal cells into decidual cells involves the existence of active mechanisms regulating chemokine expression and function.

Human and animal studies have demonstrated that the cytokine milieu in the uterus is not only different between the pregnant and non-pregnant uterus, but it differs between early and late pregnancy^6,27,28^. Indeed, implantation and early trophoblast invasion have been shown to depend on the presence of inflammatory signals necessary for the attachment of the embryo to the surface epithelium of the uterus^29^. However, once implantation is achieved, the inflammatory environment needs to be reversed into an anti-inflammatory state in order to prevent maternal rejection of the embryo^30,31^. The signals regulating this shift at the implantation site have not yet been defined.

Human chorionic gonadotropin (hCG) is one of the earliest hormones produced by the blastocyst and has potent immune modulatory effects, especially in relation to T cells^32–34^, B cells and dendritic cells^35,36^. hCG has been suggested to induce the immune modulatory changes in the phenotype and function of immune cells by either a direct pathway that involves the direct binding of hCG to its receptor on T and B cells^34,37^, or indirect pathways by inducing changes in regulatory cell populations such as dendritic cells^38–40^ or decidual/stromal cells. The interaction between hCG and stromal cell differentiation and immune regulatory function is unknown.

Several of the modifications taking place in the uterus in preparation for and during pregnancy are epigenetic modifications in response to local and systemic signals. The Polycomb-group (PcG) proteins make up the two Polycomb repressor complexes, PRC1 and PRC2^41^ and are major regulators of cell differentiation. Each complex is composed of known core proteins, which represent the evolutionary conserved components of the complex^42–45^. Fine-tuning of the different components of each PRC has been observed in different cell types and in different cellular states^46^. In humans, PRC1 is composed of core proteins Bmi-1/Mel-18, CBX, RING1, and PHC while PRC2 is composed of EZH2, EED, and SUZ12. Multiple isoforms exist for each PcG protein and therefore, depending on the cell type and cellular state, the components of each PRC vary. PRCs suppress gene transcription through binding to the chromatin at the polycomb responsive elements (PRE) and promoting methylation^47–49^. EZH2 provides the methyltransferase activity on histone lysine residues that is essential for inducing transcriptional repression and stable gene silencing. Although it has been shown that the PcG proteins play an important role in the differentiation and function of decidual cells, the factors controlling these epigenetic modifications have not been elucidated. Previously, Nancy *et al*.^50^ reported in mice that *CXCL9* and *CXCL10*, two important chemokines, are transcriptionally silenced during decidualization in the mouse uterus. This happened in association with promoter addition of tri-methyl histone H3 lysine 27 (H3K27me3), a repressive histone mark generated by polycomb repressive complex 2 (PRC2). H3K27me3 is an epigenetic modification to the DNA packaging protein Histone H3 and constitutes a mark that indicates the tri-methylation to the 27th lysine residue of the histone H3 protein. This tri-methylation is associated with the downregulation of nearby genes via the formation of heterochromatic regions^51^.

The objective of this study was to characterize the mechanisms associated with the regulation of the chemokine *CXCL10* expression during the process of stromal-decidual differentiation. We tested the hypothesis that the trophoblast signal hCG, is responsible for the suppression of pro-inflammatory cytokines, specifically *CXCL10* expression by uterine stromal cells by regulating H3K27me3 histone modifications. We report the identification of a specific site at the promoter region of *CXCL10* modified by H3K27me3 and show that hCG-induced H3K27me3 modification requires the recruitment of the PRC2 member EZH2. Furthermore, we describe the clinical correlation between circulating hCG and *CXCL10* levels during early gestation in normal pregnancies.

## Results

### Decidual stromal cells (DSCs) secrete a specific cytokine profile that results in decreased CD8 T cell migration compared to stromal cells

**Figure 1:**
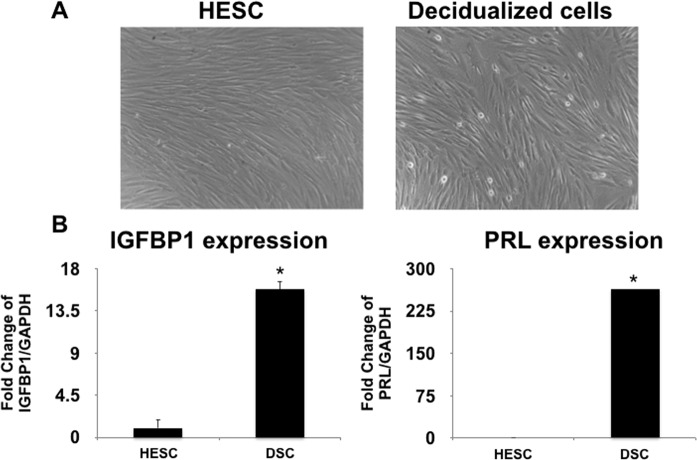
*In vitro* model of decidualization. A human endometrial stromal cell line (HESCs)^52^ was decidualized with 10 nM estradiol, 1 uM medroxyprogesterone acetate, and 500 uM 8-bromo cyclic AMP (cAMP) for 7 days. (**A**) Morphological changes associated with the decidualization process. The left panel shows the untreated HESCs, while the right panel shows the decidualized cells (DCS) after treatment with estradiol, medroxyprogesterone acetate, and 8-bromo cyclic AMP. (**B**) Increased expression of Prolactin and IGFBP1 in decidualized cells (DSC) compared to HESC. Data presented as mean ± SD and are shown for 6 independent experiments with each done in triplicate. *p < 0.001.

**Figure 2:**
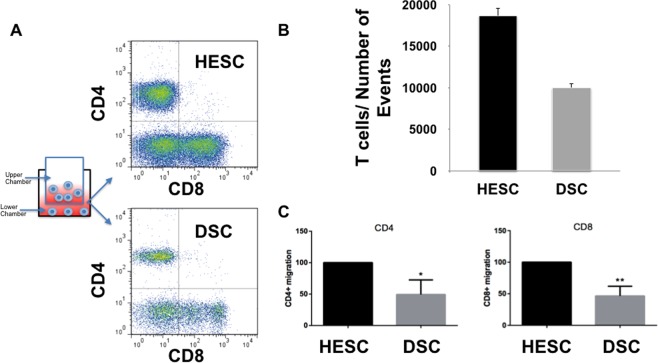
Recruitment of immune cells by stromal cell- derived factors. Supernatants from HESC and decidualized HESC (DSC) cells were collected after 5 days of culture (equal confluence) and used as conditioned media (CM) for testing their effect on the migration of peripheral blood mononuclear cells (PBMC) using a two- chamber migration assay. PBMCs were added to the upper chamber for 24 h. The migrated cells (lower chamber) were collected, phenotyped, and counted by flow cytometry. (**A**) Representative figure of the different cell types collected in the lower chamber recruited by HESC and DSC’s conditioned media. Both CD4 and CD8 T cells migrated towards HESC CM and DSC CM. (**B**) Quantification of the total number of T cells recruited towards conditioned media obtained from HESC and DSC. Data shown are for 6 independent experiments with each group done in triplicate. *p < 0.001. (**C**) Flow Cytometry Analysis of CD4 and CD8 T cells. Percentage of CD4 and CD8 T cells recruited by DSC CM compared to HESC CM. DSC CM recruits significantly lower number of CD4 and CD8 T cells compared to HESC CM. Data presented as mean/SEM and are for 6 independent experiments with each group done in triplicate. *p < 0.05. **p < 0.01.

Our first objective was to determine the capacity of human endometrial stromal cells versus decidual cells to recruit immune cells and to characterize their chemokine profile. To achieve this objective, we used an *in vitro* model consisting of a telomerase immortalized human endometrial stromal cell line (HESCs) which can undergo *in vitro* differentiation into decidual cells (DSCs) by continued treatment with estrogen, progesterone and cAMP as previously reported and described in the Material and Methods^52^. In short, HESCs were exposed to estrogen, progesterone and cAMP for 5–9 days and the differentiation of HESCs into DSCs was confirmed by morphologic changes such as enlarged, rounded morphology compared to the elongated spindle-shaped fibroblastic stromal cells (Fig. 1A) and the increased expression of Prolactin and IGFBP-1 (Fig. 1B)^52^. Afterwards, we evaluated the capacity of these cells to recruit immune cells by using a 5 µM trans-well two-chamber migration assay where conditioned media (CM) collected from HESCs or DSCs (see M&M for preparation of CM) were added to the bottom chamber as chemoattractant and peripheral blood mononuclear cells (PBMCs) (350,000 cells/well) were added to the upper chamber. After 24 h, all the cells that migrated into the lower chamber were collected and the number of T cells (CD4 and CD8) was evaluated by flow cytometry. We observed a significantly higher number of T cells present in the chambers containing HESCs’ CM (18,543 ± 115 T cells) compared to the chambers containing CM from DSCs (10,007 ± 1654 T cells) (p < 0.05) (Fig. 2B). The proportion of recruited CD4 and CD8 T cells were significantly reduced in the chambers containing CM from DSCs compared to the chambers containing HESCs’ CM (Fig. 2A,C)

These findings suggest that HESCs are highly effective in recruiting T cells; however, differentiation into DSCs is associated with a significant drop in their recruitment capacity.

Cytokines and chemokines are the main mediators of immune cell recruitment; consequently, we evaluated, using Luminex analysis, whether there were differences in the cytokine and chemokine profile between HESC and DSCs that could explain the observed differential capacity to recruit T cells between these two cell types. Table 1 summarizes the cytokine/chemokine profile from supernatants obtained from these two cell types. HESCs are characterized by the secretion of pro-inflammatory cytokines, IL6, IL-12, IL-17, IFN-γ and chemokines IL-8, G-CSF CXCL-10, MIP-1α, VEGF and CCL5 (RANTES) (Table 1); however, upon differentiation into DSC cells, we observed a significant reduction in the secretion levels of these pro-inflammatory cytokines and chemokines (Table 1).

**Table 1:** Cytokines/Chemokines expression levels.

Cytokine	HESC	DSC*	HESC + hCG*
CXCL10	307.13 +/− 34.45	30.18 +/− 3.76 (<0.05)	16.51 +/− 9.38 (<0.05)
GRO-alpha	484.59 +/− 90.62	0 (<0.05)	0 (<0.05)
IL-12	33.64 +/− 4.68	6.25 +/− 0.58(<0.05)	15.89 +/− 3.15 (<0.05)
IL-17	10.27 +/− 1.26	3.90 +/− 1.74(<0.05)	2.26 +/− 0.1 (<0.05)
G-CSF	18.72 +/− 3.02	4.63 +/− 0.91 (<0.05)	0.83 +/− 1.05 (<0.05)
IFN-γ	22.49 +/− 1.5	0 (<0.05)	0 (<0.05)
IL-8	18650.72 +/− 13471.22	438.30 +/− 141.79 (<0.05)	447.75 +/− 90.90 (<0.05)
MCP-1	2229.11 +/− 517.63	1157.14 +/− 183.77 (NS)	29.2 +/− 1.73 (<0.05)
MIP-1 alpha	0.44 +/− 0.06	0 (NS)	0 (NS)
RANTES	429.68 +/− 30.10	48.44 +/− 4.57 (<0.05)	10.52 +/− 4.10 (<0.05)
VEGF	127.15 +/− 20.92	22.85 +/− 1.4 (<0.05)	175.14 +/− 29.13 (NS)

*Differential expression in relation to HESC.

*CXCL10* is a potent chemoattractant for CD8 + T cells and is highly expressed in the supernatant of HESCs but its expression is dampened upon differentiation into DSC; hence, we hypothesized that the decrease in migration of CD8 + T cells with the DSCs CM might be due to the inhibition of *CXCL10* expression after decidualization. Accordingly, we sought to elucidate the potential mechanisms responsible for the regulation of *CXCL10* expression in HESCs/DSCs and its impact on T cell recruitment.

### hCG/cyclic AMP pathway regulates *CXCL10* expression during cellular differentiation

**Figure 3:**
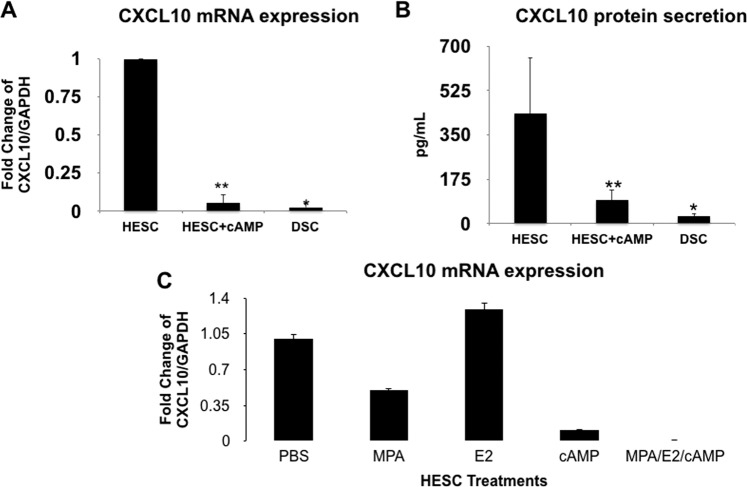
Regulation of *CXCL10* expression in stromal versus decidual cells. (**A**) Effect of cAMP and decidualization on *CXCL10* mRNA expression. HESC were treated with 500 uM 8-bromo cAMP alone or decidualized with 10 nM estradiol, 1 uM medroxyprogesterone acetate, and 500 uM 8-bromo cyclic AMP for 7 days. Note the significant inhibition of *CXCL10* mRNA expression by cAMP in HESC, similar to the decrease observed in the decidualized cells (DSC) *p < 0.05. Data presented as mean ± SD and are from 3 independent experiments with each group done in triplicate. (**B**) Quantification of *CXCL10* protein expression in HESC treated with cAMP or decidualized with estrogen, progesterone and cAMP. cAMP treatment and decidualized cells (DSC) are associated with decreased *CXCL10* protein expression. *p < 0.001. **p < 0.05. Data presented as mean ± SD and are from 3 independent experiments with each group done in triplicate. (**C**) Effect of estrogen, progesterone and cAMP on *CXCL10* mRNA expression. HESC were treated with 10 nM estradiol, 1 uM medroxyprogesterone acetate (MPA), and 500 uM 8-bromo cAMP individually or under the combination of estradiol, MPA and cAMP for 7 days. *CXCL10* mRNA was determined by qPCR. Note that only cAMP has a major effect on *CXCL10* mRNA expression. Data presented as mean ± SD and are from 3 independent experiments with each group done in triplicate.

In order to determine whether the process of differentiation from stromal cells into decidua cells is associated with the changes on *CXCL10* expression, we evaluated *CXCL10* mRNA levels in HESCs before and after differentiation into DSCs. Similar to the findings with the secreted protein, the process of decidualization is associated with transcriptional inhibition of *CXCL10*, demonstrated by decreased levels of *CXCL10* mRNA in DSCs, which correlated with lower levels of secreted protein expression compared to the HESCs (Fig. 3A,B). Since estrogen and progesterone are known to be the main hormones involved in the process of decidual differentiation; we first tested the effect of either estrogen or progesterone on *CXCL10* expression by stromal cells. Interestingly, neither estrogen nor progesterone were responsible for the modulatory effect on *CXCL10* expression levels during decidualization (Fig. 3C). However, we did observe a significant inhibitory effect on *CXCL10* mRNA and protein expression following cAMP treatment, the other major mediator of decidualization^53^ (Fig. 3A–C); suggesting that factors that could activate cAMP might have modulatory effects on *CXCL10* expression.

hCG is one of the early factors secreted by the trophoblast and is known to have immune modulatory effects during pregnancy, especially with regards to the recruitment of regulatory T and B cells^33,34^. The cellular effects of hCG are mediated through the LH receptor (LHR), a G-protein coupled receptor, and ligation of the receptor results in an increase in intracellular cAMP, its secondary messenger whose resulting protein cascades affect target gene expression^54^. Therefore, we hypothesize that hCG, though the induction of cAMP could inhibit recruitment of CD8^+^ T cells by suppressing *CXCL10* expression in endometrial stromal cells. To test this hypothesis, we first evaluated the overall effect of hCG on cytokine/chemokine expression by HESCs. As shown in Table 1, column 3, hCG treatment had a notable inhibitory effect on HESCs’ cytokine and chemokine expression profile, similar to the one observed with the process of differentiation into decidual cells (Table 1).

**Figure 4:**
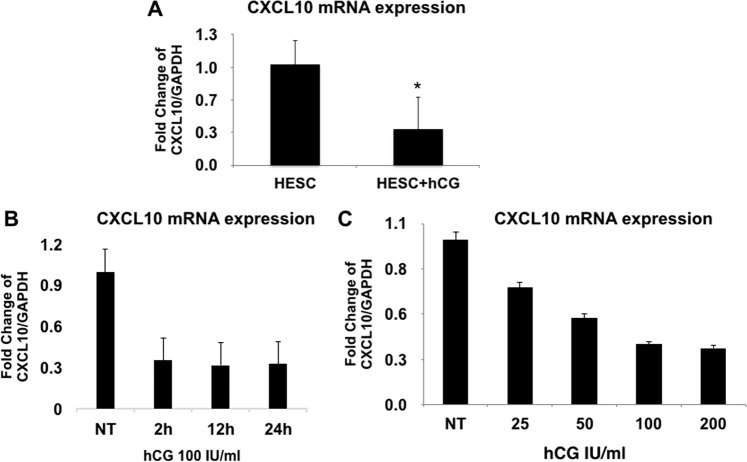
Regulation of *CXCL10* expression by hCG in human endometrial stromal cells (HESC). (**A**) HESC were treated with hCG 100 IU/ml for 24 h and *CXCL10* mRNA expression was determined by qPCR. Note the significant decrease of *CXCL10* mRNA expression after hCG treatment. Data presented as mean ± SD and are for 6 independent experiments with each group done in triplicate *p < 0.005. (**B**) Time response to hCG treatment (100 IU/ml). HESC were treated with hCG (100 IU/ml) for 2, 12 and 24 h and *CXCL10* mRNA expression was determined by qPCR. *CXCL10* mRNA expression decreased within 2 hours of hCG treatment and remained low up to 24 hours of treatment. (**C**) Dose response to hCG treatment. HESC were treated with increasing concentrations of hCG and *CXCL10* mRNA expression was determined by qPCR. *CXCL10* mRNA expression in HESC decreased in a dose dependent manner.

Next, we characterized the potential transcriptional regulatory role of hCG on HESC’s *CXCL10* expression by treating HESCs with hCG and determining *CXCL10* mRNA expression. As shown in Fig. 4, HESCs treated with hCG revealed decreased *CXCL10* mRNA expression (Fig. 4A) and this effect was dose and time dependent, further supporting the specificity of its effect (Fig. 4B,C). The inhibitory effect observed with hCG resembles the one determined with cAMP (Fig. 3).

### hCG/cyclic AMP pathway is an epigenetic regulator of CXCL10 expression by inducing histone methylation

**Figure 5:**
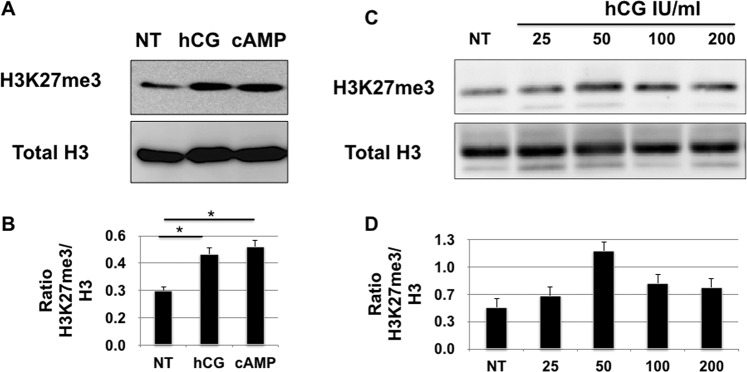
Regulation of H3K27me3 expression by hCG and cAMP in human endometrial stromal cells. (**A**) hCG and cAMP induce H3K27me3 in HESC. HESC were treated with hCG (100 IU/mL) or cAMP (500 uM) and H3K27me3 expression was determined by Western blot analysis. Representative western blot analysis showing hCG and cAMP enhanced H3K27me3 expression in HESCs. (**B**) Quantification of the western blot analysis showed in figure A. Data is presented as Relative Intense Units (RIU) as an average of 3 independent experiments. *p < 0.001. (**C**) Dose response effect of hCG on H3K27me3 expression. HESC were treated with increasing concentrations of hCG for 24 h followed by the determination of H3K27me3 expression by Western blot. hCG induces a dose dependent increase on H3K27me3 expression. Data is representative of 3 independent experiments done in triplicate. (**D**) Quantification of the western blot analysis showed in figure C. Data is presented as Relative Intense Units (RIU) as an average of 3 independent experiments.

Epigenetic modifications, such as histone methylation, play a central role in the regulation of cytokines and chemokines expression^55^; therefore, we hypothesized that the hCG/cAMP pathway may silence the promoter of *CXCL10* (the human gene that codes for *CXCL10*) by histone methylation. As indicated above, H3K27me3 modification is associated with gene shutting down target gene transcription^56,57^. First, we determined whether the hCG/cAMP pathway could affect global H3K27 methylation by treating HESCs with hCG or cAMP for 2 h and evaluating H3K27me3 modification by western blot analysis. As shown in Fig. 5A, HESCs cells treated with either hCG or cyclic AMP showed increased levels of H3K27me3 modification when compared to non-treated cells (Fig. 5A,B). Furthermore, when we expose HESCs to increasing concentrations of hCG, we observed a dose dependent increase on H3K27me3 modification (Fig. 5C,D). Total H3 was not affected by any of these treatments, confirming that the effect is associated with increasing methylation of H3K27 (Fig. 5A–D).

**Figure 6:**
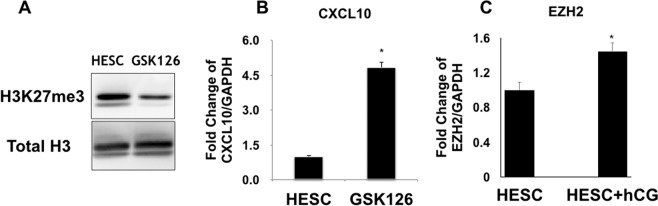
Regulation of EZH2 expression by hCG in human endometrial stromal cells. (**A**) Treatment of HESC with the EZH2 inhibitor GSK126 (2μM) decreases H3K27me3 expression. HESC were treated with GSK126 (2μM) for 24 h followed by the evaluation of H3K27me3 expression by Western blot analysis. Note the decrease of H3K27me3 expression in the presence of GSK126. Representative Western blot analysis from 6 independent experiments. (**B**) Treatment of HESC with the EZH2 inhibitor GSK126 (2μM) increases *CXCL10* mRNA expression. HESC were treated with GSK126 (2μM) for 24 h followed by the evaluation of *CXCL10* mRNA expression by qPCR. Treatment with GSK126 is associated with significant increase in *CXCL10* mRNA expression. *p < 0.002. Data shown as mean + SD and are for 6 independent experiments. (**C**) Effect of hCG on EZH2 mRNA expression. HESC were treated with hCG (100 IU/mL) for 24 h and *CXCL10* mRNA expression was determined by qPCR. hCG treatment enhances EZH2 mRNA expression in HESC. *p < 0.05. Data shown as mean + SD and are for 3 independent experiments.

Next, we sought to determine the molecular mechanism of how hCG mediates H3K27me3 modification. The PRC2 complex is the main regulator of H3K27me3 modification through the specific histone methyltransferase, EZH2. EZH2 catalyzes the methylation of Histone 3 at Lysine 27 (K27)^45^. Hence, we evaluated EZH2 in the regulation of H3K27me3 in HESC by using an inhibitor for EZH2, GSK126. Thus, DSCs, which express high levels of H3K27me3 and low levels of *CXCL10*, were treated with GSK126 (2μM) for 24 h followed by evaluation of *CXCL10* mRNA expression and H3K27me3 by western blot analysis. Treatment of DSCs with GSK126 decreased H3K27me3 modification, as determined by western blot analysis (Fig. 6A); and, interestingly, correlated to enhanced *CXCL10* mRNA expression (5-fold) (Fig. 6B). We then determined whether hCG effect on H3K27me3 could be mediated through the regulation of EZH2 expression. Thus, HESCs were treated with hCG and EZH2 mRNA expression was determined by qPCR. We observed that hCG treatment had a modest, but significant effect on EZH2 mRNA expression (1.5-fold increase) (Fig. 6C). These findings further suggest an epigenetic regulation of *CXCL10* expression in endometrial stromal cells.

### Specific Binding of H3K27me3 to the *CXCL10* promoter in human decidual tissues

**Figure 7:**
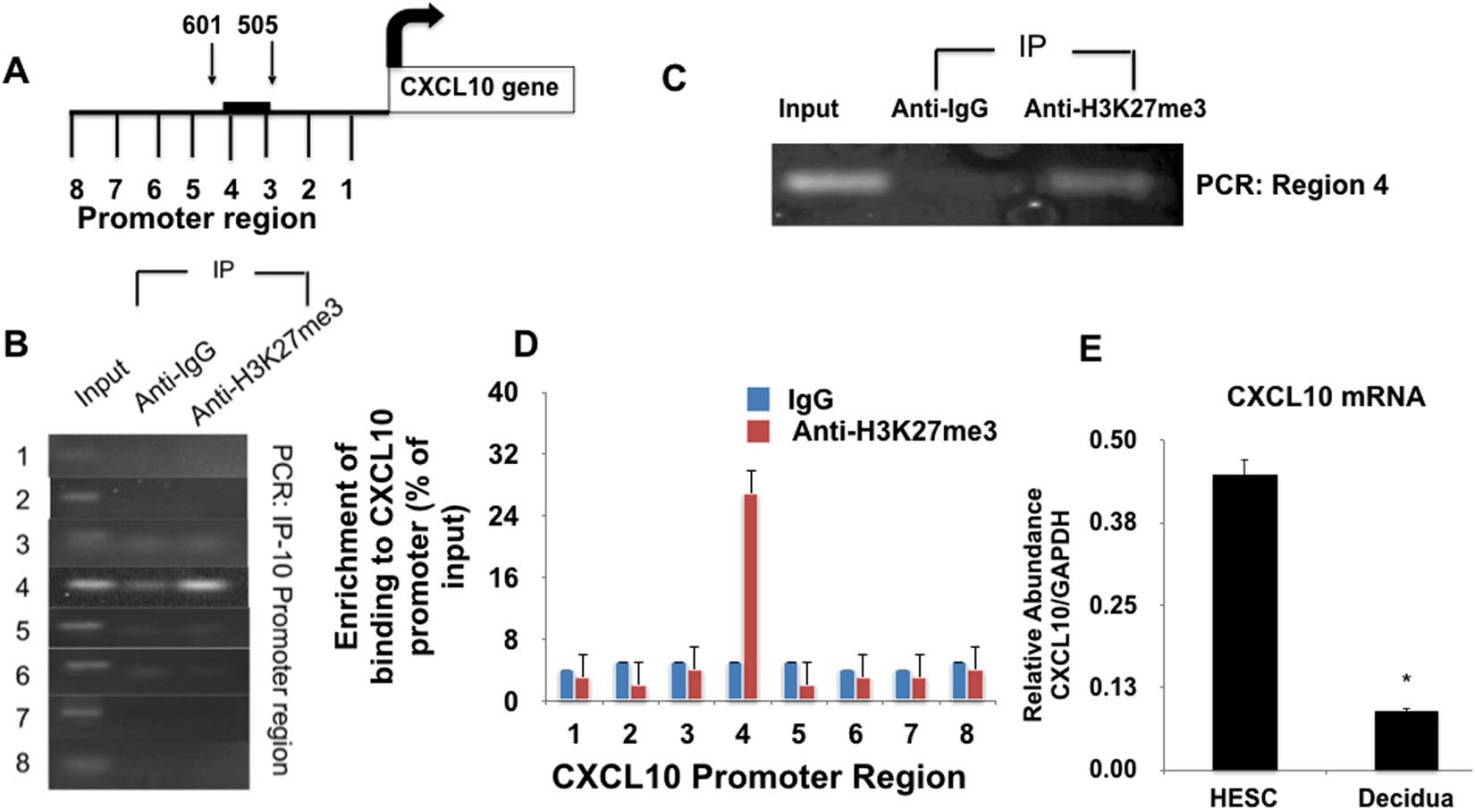
Identification of a novel site of epigenetic regulation in the *CXCL10* promoter in term human decidual tissue. (**A**) Diagram of the *CXCL10* promoter region. Eight specific primers were designed to cover the full length of the *CXCL10* promoter region (IP-10 = *CXCL10*). (**B**) Chromatin immunoprecipitation was performed on human decidual samples collected from normal term non-labored placentas delivered by cesarean delivery. Decidual samples were crosslinked, sonicated and immunoprecipitated using an anti-H3K27me3 antibody. The DNA enriched with the H3K27me3 was purified and amplified by qPCR for the different regions of the *CXCL10* promoter. Immunoprecipitation of H3K27me3 and PCR amplification of the DNA sequence bound by the histone demonstrated a strong band at Region 4 (505–601 base pairs upstream of the *CXCL10* gene) in the promoter region of *CXCL10* gene. No bands were detected at regions 1–3 or 5–8, indicating that the histone mark was not enriched in those regions. (**C**) PCR amplification of the DNA from Region 4 of the *CXCL10* promoter that was bound by the H3K27me3 after immunoprecipitation using an anti-H3K27me3 antibody. Representative figure of 6 independent decidual samples. (**D**) Quantification of the enrichment of the DNA sequence in the different regions of the *CXCL10* promoter. Note that only Region 4 reveled binding to the H3K27me3 after ChIP qPCR. n = 6 human decidual tissue samples. (**E**) Relative *CXCL10* mRNA expression levels from decidual tissue samples in relation to stromal cells. *p < 0.05. n = 6 human tissue samples.

So far, we demonstrated *in vitro*, using cell lines, that hCG treatment affected H3K27me3 modification levels and correlates with *CXCL10* mRNA and protein expression. Our next objective was to determine whether these findings are also present in freshly collected decidual tissue that has not undergone any *in vitro* manipulation. The main justification to use the whole decidual tissue is to avoid the potential non-specific effect of cell isolation and cell culture. Thus, decidual samples were obtained from term, non-labored, uncomplicated pregnancies undergoing scheduled cesarean delivery. Using these freshly collected decidual samples we then evaluated whether H3K27me3 modification is present in the tissues and if is responsible for the suppression of *CXCL10* gene expression. First, we determined the enrichment of this histone mark on the *CXCL10* promoter by performing chromatin immunoprecipitation using an anti-H3K27me3 antibody on freshly collected decidual tissue. After immune precipitation with the anti-H3K27me3 antibody we evaluated the enrichment of the histone mark in the *CXCL10* promoter by using eight pairs of primers designed to span the 1000 bp upstream of the human *CXCL10* gene. In human term uncomplicated non-labor decidua, we observed enrichment of H3K27me3 in Region 4 (505–601 base pairs upstream) of the human *CXCL10* promoter (Fig. 7A,B). There was no appreciable enrichment in regions 1–3, or 5–8 (Fig. 7A,B). These findings were validated and quantified in six human decidua specimens (Fig. 7C,D). Only in region 4 we are able to obtain quantitative enrichment of H3K27me3 in the *CXCL10* promoter (Fig. 7D). Figure 7C shows a representative DNA gel of the PCR product after chromatin immunoprecipitation demonstrating enrichment of the H3K27me3 to Region 4 of the *CXCL10* promoter in human decidua samples. The presence of the H3K27me3 mark at region 4 of the *CXCL10* promoter correlates with low levels of *CXCL10* mRNA expression in decidual tissue compared to stromal cells (Fig. 7E). Stroma cells are used as positive control for CXCL10 expression.

### hCG regulates the Binding of H3K27me3 to the *CXCL10* promoter in human stromal cells

**Figure 8:**
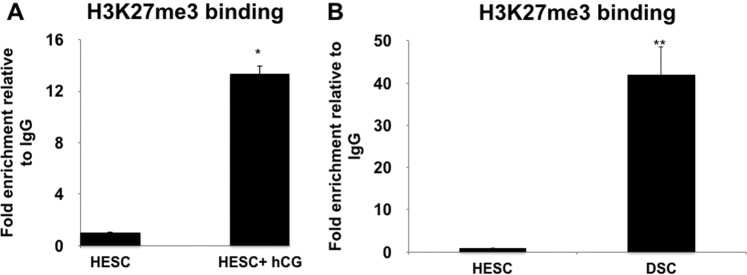
hCG regulates *CXCL10* expression and secretion by increasing H3K27me3 histone enrichment in Region 4 of the *CXCL10* promoter. (**A**) HESC were treated with either vehicle or hCG (100 IU/ml). The cells were crosslinked, sonicated and immunoprecipitated using an anti-H3K27me3 antibody. The DNA enriched with the H3K27me3 was purified and amplified for Region 4 of the *CXCL10* promoter. The results were quantified by ChIP qPCR. Note the significant enrichment of Region 4 bound to H3K27me3 in cells treated with hCG. *p < 0.05. Data shown are for 3 independent experiments. (**B**) Enrichment of the H3K27me3 mark in the *CXCL10* promoter from HESC undergoing decidualization. HESC were decidualized with estrogen, progesterone and cAMP for 5 days. Chromatin immunoprecipitation was performed in HESC and decidualized stromal cells using an anti-H3K27me3 antibody. The enrichment was quantified using ChIP qPCR. Note the significant enrichment of Region 4 bound to H3K27me3 in decidualized cells compared to HESC. **p < 0.002. Data shown for 3 independent experiments.

Having identified Region 4 as the site for H3K27me3 modification at the *CXCL10* promoter in decidual tissues, our next objective was to determine whether hCG could promote this modification. Accordingly, we treated HESCs, expressing high levels of *CXCL10*, with hCG (100 IU/ml) for 2 h, followed by chromatin immunoprecipitation and PCR for enrichment of H3K27me3 mark at the *CXCL10* promoter. Our data showed a 15-fold increased enrichment of H3K27me3 modification at Region 4 of the *CXCL10* promoter in HESCs treated with hCG compared to non-treated cells (Fig. 8A). Similar as reported with decidual tissues (Fig. 7), no modifications were found in the other regions; confirming the specificity for region 4 of the *CXCL10* promoter.

We then compared whether similar effect is observed during the *in vitro* process of differentiation into decidual cells. Thus, HESC were exposed to estrogen, cAMP and progesterone for 5 days in order to induce decidualization, followed by chromatin immunoprecipitation and PCR for enrichment of the H3K27me3 mark at the *CXCL10* promoter. As shown in Fig. 8B, we confirmed that the process of differentiation from HESCs to DSCs is associated with the enrichment of the histone mark to region 4 of the *CXCL10* promoter (42-fold increase; p < 0.01) (Fig. 8B). No changes were observed in the other regions (Fig. 7B,D).

### Bacterial Lipopolysaccharide (LPS) inhibits H3K27me3 modification and induces *CXCL10* expression in term human decidual tissue through the histone demethylase JMJD3

**Figure 9:**
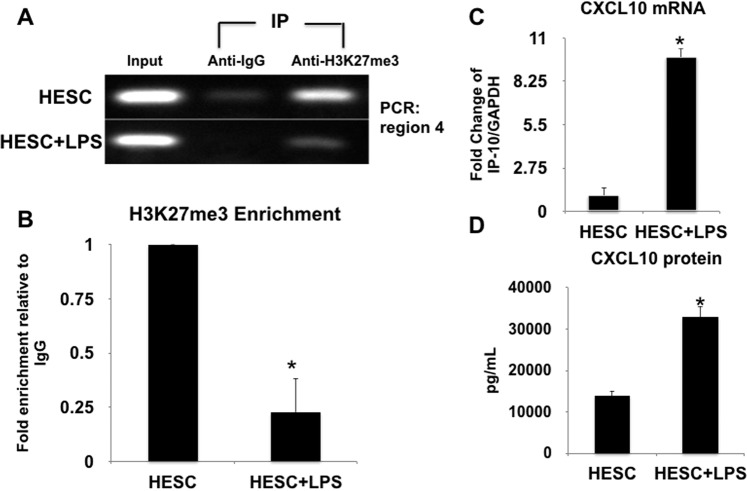
Effect of LPS on *CXCL10* expression and secretion. (**A**) Human term decidua tissues were treated with LPS (100 ng/ml) for 24 followed by chromatin immunoprecipitation using an anti-H3K27me3 antibody. The DNA enriched with the H3K27me3 was purified and amplified by qPCR for Region 4 of the *CXCL10* promoter. Note the decreased binding of the H3K27me3 to region 4 of the *CXCL10* promoter observed in cells treated with LPS compared to the control (PBS) treated group. Representative figure of 6 independent experiments. (**B**) Quantification of the enrichment of the DNA sequence of region 4 of the *CXCL10* promoter bound to H3K27me3. The enrichment was quantified using ChIP qPCR and reveled decreased enrichment in decidual samples exposed to LPS. p < 0.05. (**C**) Effect of LPS on *CXCL10* mRNA expression. Human term decidua tissue samples were treated with LPS (100 ng/ml) for 24. *CXCL10* mRNA expression was determined by qPCR. LPS treatment induces a significant increase on *CXCL10* mRNA expression in human term decidua tissues n = 6, *p < 0.05. (**D**) Human term decidua tissues were treated with LPS (100 ng/ml) for 24 followed by evaluation of *CXCL10* protein expression by Luminex. LPS induces *CXCL10* protein secretion in decidual samples. n = 6, *p < 0.001.

Our next objective was to determine the potential signals that could disrupt the regulation of *CXCL10* expression by decidual cells. Since bacterial infections are a major cause of pregnancy complications and are able to induce and inflammatory process at the maternal/fetal interface including the expression of inflammatory cytokines/chemokines such as *CXCL10*^58^, we hypothesized that bacterial signals could induce *CXCL10* expression by modifying the H3K27me3 mark and consequently promote the recruitment of maternal CD8^+^ T cells. To test this hypothesis, first we used the organ culture model of normal human term decidual tissue obtained from non-labored cesarean deliveries which, as described above, express low levels of *CXCL10* and express high levels of the H3K27me3 mark at Region 4 of the *CXCL10* promoter. Thus, decidual tissues were exposed to PBS (Control) or lipopolysaccharide (LPS, 100 ng/mL) for 24 h and evaluated for the presence of H3K27me3 mark at region 4 of the *CXCL10* promoter by chromatin immunoprecipitation using the anti-H3K27me3 antibody. In addition, we determined the expression of *CXCL10* mRNA and protein by qPCR and Luminex assay, respectively. Decidual tissue samples exposed to control PBS showed strong enrichment of the H3K27me3 mark at Region 4 of the *CXCL10* promoter (Fig. 9A), which correlates with low levels of *CXCL10* mRNA and protein expression (Fig. 9C,D). That was not the case for the decidual samples treated with LPS where we observed a decrease of the H3K27me3 mark at Region 4 of the *CXCL10* promoter (Fig. 9A). Using ChIP qPCR we quantified the level of enrichment for H3K27me3 in Region 4 of *CXCL10* promoter and confirmed the decrease of the H3K27me3 mark following LPS treatment compared to the control vehicle (Fig. 9B). Changes in the presence of H3K27me3 modification at the *CXCL10* promoter correlated with the expression levels of *CXCL10*. Indeed, our data showed that LPS treatment, by decreasing the presence of H3K27me3 modification prompted increase on *CXCL10* expression at the mRNA (Fig. 9C) and protein level (Fig. 9D). We observed a 10-fold increase in *CXCL10* mRNA expression in the LPS treated group compared to the control vehicle (Fig. 9C). Similarly, at the protein level we observed a significant increase in the concentration of secreted *CXCL10* in the LPS treated group (32,832.56 ± 1, 902.12 pg/mL) compared to control no treatment (13,886,86 ± 1,254.27 pg/mL*p < 0.05) (Fig. 9D). These findings further demonstrate that the H3K27me3 modification is present in human decidual tissue, regulating *CXCL10* expression and can be modified by signals induced by bacterial products such as LPS.

**Figure 10:**
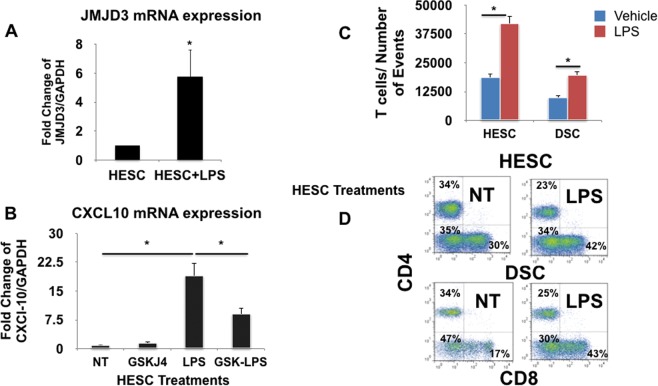
Role of JMJD3 in the regulation of *CXCL10* expression and secretion. (**A**) Expression of JMJD3 in human decidual tissue. Human term decidual tissues were treated with LPS (100 ng/ml) and JMJD3 expression was determined by qPCR. LPS treatment enhances JMJD3 mRNA expression. n = 6, *p < 0.05. (**B**) Inhibition of JMJD3 function with the inhibitor GSKJ4 prevents LPS-induced *CXCL10* expression. Human term decidual tissues were treated with LPS (100 ng/ml) in the presence or absence of GSKJ4, an inhibitor of JMJD3 for 24 h. *CXCL10* mRNA expression was determined by qPCR. The presence of GSKJ4 inhibits LPS induced *CXCL10* expression. *p < 0.05. (**C**) Quantification of the total number of T cells recruited towards conditioned media obtained from HESC and DSC in the presence or absence of LPS treatment (100 ng/ml). Transwell migration assays were performed using peripheral blood mononuclear cells and conditioned media from HESC and DSC treated with LPS or vehicle (control). LPS treatment is associated with increased number of T cells collected in the lower chamber (recruitment chamber). n = 6 independent experiments. Each group was done in triplicates. *p < 0.001. (**D**) Flow Cytometry Analysis of CD4^+^ and CD8^+^ T cells. Percentage of CD4^+^ and CD8^+^ T cells recruited by DSC CM in relation to HESC CM was determined by flow cytometry. CD4^+^ and CD8^+^ T cells were identified in the Transwell migration assays. Representative flow cytometry from four independent experiments.

Next, we evaluated the mechanism how LPS decreases the H3K27me3 mark at Region 4 of the *CXCL10* promoter. We screened for changes in the expression of known H3K27me3 histone demethylases such as KDM6A and JMJD3^59^ in response to LPS treatment and found that the mRNA expression of JMJD3, a histone demethylase specific to the methylated histone H3K27me3, was affected by LPS treatment. Thus, when decidual organ cultures were exposed to PBS (Control) or LPS (100 ng/mL) for 24 h and evaluated JMJD3 mRNA expression, decidual organ cultures exposed to LPS showed a 3-fold increase in JMJD3 mRNA expression compared to the PBS control (p < 0.05) (Fig. 10A). The increase in JMJD3 expression correlated with LPS-induced decrease of H3K27me3 modification from the *CXCL10* promoter (Fig. 9A,B) and increased *CXCL10* mRNA expression and protein secretion (Fig. 9C,D).

To further confirm the role of JMJD3, we treated the decidual organ cultures with GSK-J4, a specific JMJD3 inhibitor, in the presence or absence of LPS and then examined *CXCL10* expression levels. As shown in Fig. 10B, pre-treatment of decidual organ cultures with the GSK-J4 reversed the effect of LPS on *CXCL10* mRNA expression. While LPS treatment induced a 19-fold increased on *CXCL10* mRNA expression, the presence of GSK-J4 significantly reduced LPS-induced *CXCL10* mRNA expression (Fig. 10B; p < 0.05). GSK-J4 treatment alone did not affect *CXCL10* mRNA expression (Fig. 10B). This data demonstrates that infection can induce a strong and quick inflammatory response by decreasing the H3K27me3 modification through the induction of the expression of the histone demethylase JMJD3.

### LPS removal of H3K27me3 modification in DSCs results in increased recruitment of CD8** +** T cells

Finally, we determined whether LPS, by decreasing the H3K27me3 modification and enhancing *CXCL10* expression could affect the capacity of DSCs to recruit T cells. Thus, we used the same trans-well migration assay as described in Fig. 2. PBMC were added to the upper chamber and then exposed to CM in the lower chamber obtained from: (1) DSCs treated with vehicle (control) or (2) DSCs treated with LPS (100 ng/mL) for 48 h. This concentration of LPS has no detrimental effect on DSCs^60–63^. As shown in Fig. 10C, LPS treatment of HESC and DSCs enhances the recruitment of T cells. Indeed, CM from DSCs treated with LPS attracted more T cells than CM from non-treated DSCs (11,832 ± 821.7 vs 1905 ± 165, p < 0.05). A high percentage of the recruited cells were CD8 + and CD4 + T cells (Fig. 10D).

### Inverse correlation between hCG and *CXCL10* levels in early pregnancy

**Figure 11:**
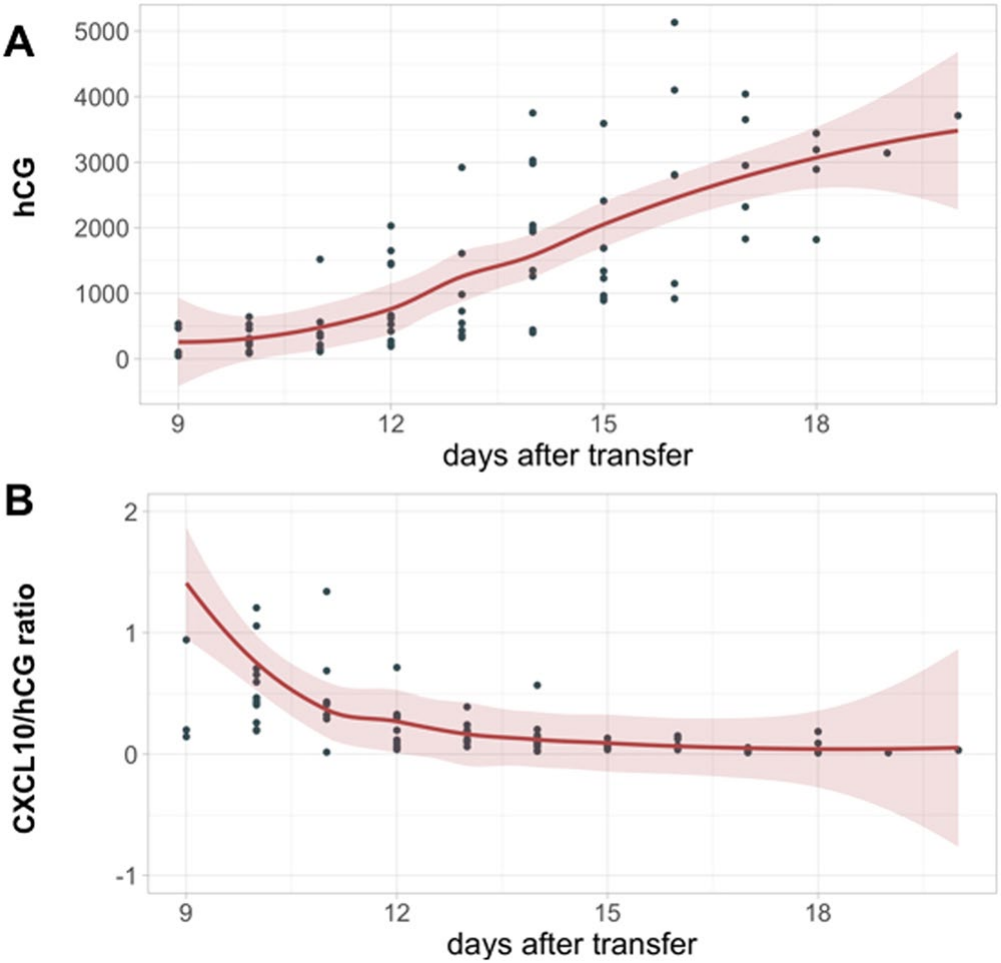
hCG and CXCL10 expression ratio throughout early gestation. Circulating levels of hCG and *CXCL10* were measured using ELLA assay in serum samples collected from normal pregnant women during the first 4 weeks of pregnancy. (**A**) hCG is detected in the serum as early as 9 days after embryo transfer and shows a steady increase in the following days. (**B**) Ratios of *CXCL10* and hCG across gestational age were determined in blood samples collected from IVF pregnancies. The line represents the mean estimation by LOWESS and the colored areas the 95% confidence intervals of the mean estimation. *CXCL10*:hCG ratios decreased in normal pregnancies in relation to gestational age.

So far as we have described *in vitro*, there is an inverse correlation between hCG and *CXCL10* expression. Since *CXCL10* expression levels has been shown to be higher during human embryo implantation^64^ and subsequently decreased throughout the pregnancy^65^, we explored whether we could detect and determine in the serum of pregnant women a potential correlation between circulating hCG and CXCL-10 in the first 8 weeks of pregnancy, a period when hCG production increases as result of successful placentation. Although circulating cytokines can be affected by multiple processes taking place in the body, it is the only non-invasive source for evaluating some of the changes occurring during pregnancy^66^. To achieve this objective, we recruited an IVF cohort where we were able to monitor the day of implantation and collect samples from the earliest stages of pregnancy (between 3 and 6 weeks of gestation) and measured hCG and *CXCL10* in the serum of these pregnant women throughout the first trimester of pregnancy. We detected *CXCL10* in the serum of pregnant women as early as 9 days after embryo transfer and it serum levels remained constant during the subsequent days (SFig. 3). Circulating hCG levels gradually increased as pregnancy progresses (Fig. 11A); interestingly, when we look at the ratio between *CXCL10* and hCG we observed an inverse correlation as the pregnancy progressed (Fig. 11B). This is a noteworthy association since *CXCL10* is known to be high during implantation and then silenced to prevent the recruitment of T cells^64^.

## Discussion

In this study, we describe the epigenetic role of hCG in the regulation of *CXCL10* in the human decidua. We report for the first time, the identification of a specific site for the repressive histone mark H3K27me3 in the promoter of the human CXCL10gene (Region 4, 505–601 kb upstream). Furthermore, we describe the role of hCG as a regulator of *CXCL10* expression by controlling the H3K27me3 mark through the expression of the histone methyltransferase, EZH2. We show that this mark can then be modified by bacterial products leading to an increase in *CXCL10* expression and enhancement of the recruitment of pro-inflammatory CD8 + T cells. The modification of H3K27me3 mark is mediated by JMJD3, a histone demethylase. Our studies show an active epigenetic crosstalk between the trophoblast and decidua that results in chemokine suppression. These findings are relevant because they describe a novel mechanism of fetal-maternal anti-inflammatory regulation that can be disrupted during infection, potentially leading to inflammation and preterm birth.

One important issue to recognize is that not all inflammation in pregnancy is detrimental. The correct timing of inflammation is essential to a healthy pregnancy. For example, it is well established that implantation in the first trimester is a pro-inflammatory event^67–71^ as is labor at the end of the third trimester^72–75^. On the other hand, the second trimester of pregnancy is characterized by an anti-inflammatory profile^31,76^. In pathological states, shifts towards a pro- inflammatory profile before term are associated with preterm birth^77–83^. Therefore, tight regulatory control of inflammation between the fetus/trophoblast unit and the decidua is critical in preventing preterm birth.

*CXCL10* (IP-10) is a 77 amino acid, 10kDA protein that belongs to the CXC family of chemokines^84^, and has been shown to have pleiotropic biological activities^23^. *CXCL10* is the most potent chemoattractant for cytotoxic CD8 + T cells^85^ and elevated levels of *CXCL10* in maternal serum, amniotic fluid, and neonatal cord blood have been associated with infection and preterm birth^21,86–88^. In the mouse, it was described e*x vivo* that the promoter region of the chemokines *CXCL9* and *CXCLl10* revealed elevated levels of the repressive histone mark H3K27me3 in DSCs versus myometrial cells, which was then confirmed *in vivo*^26^. Methylation of histone 3 at Lysine 27 (K27) is one of the most well-described epigenetic modifications. When H3K27 is trimethylated to H3K27me3, it is tightly associated with its target gene promoter, inactivating the promoter, and consequently shutting down target gene transcription^50,56,57^. These findings suggest that epigenetic modifications of stromal/decidual cells are critical regulators of these chemokines and consequently of the immune regulation at the maternal fetal interface.

**Figure 12:**
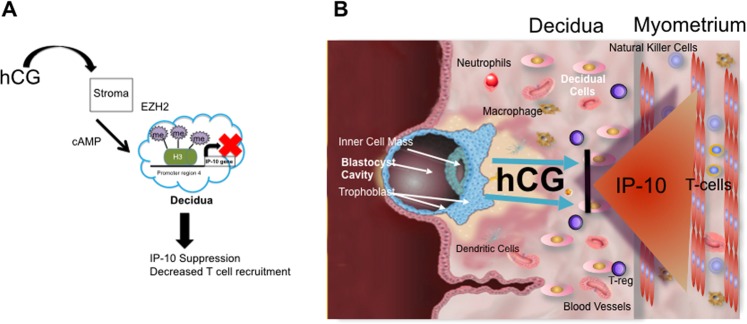
Regulation of *CXCL10* by hCG and its relevance during pregnancy. (**A**) hCG, through cAMP enhances the expression of EZH2 increasing H3K27me3 histone methylation which binds to region 4 of *CXCL10* promoter region and consequently inhibiting *CXCL10* expression. (**B**) Trophoblast derived hCG inhibits *CXL10* expression by decidual cells preventing T cell recruitment.

*CXCL10* is a potent pro-inflammatory chemokine whose regulation appears to be important during pregnancy. Human cohort studies have shown that not only does *CXCL10* expression increase with normal term labor (as do other inflammatory cytokines), but importantly, elevated levels of *CXCL10* are associated with preterm birth and chorioamnionitis, suggesting that *CXCL10* suppression in the decidua is necessary for the normal course of pregnancy^21,87,89^. Recently, the notion of a decidual clock has been proposed, wherein “timing of labor” is dependent on the suppression and release of certain elements at the correct timepoint during pregnancy. In the beginning of pregnancy, suppressive factors function to maintain the pregnancy and prevent miscarriage. The suppression is then released at the end of pregnancy and allows labor to occur^90^. Our data support this theory of the decidual clock by describing the regulation of *CXCL10* in the decidua. By secreting hCG in the beginning of the pregnancy, the fetal/placental unit works to “set the timing” on the decidual clock, to suppress inflammatory mediators such as CXCL-10. Based on our data, we propose that hCG is the fetal anti- inflammatory factor of pregnancy (Fig. 12).

Our data support hCG’s anti-inflammatory and tolerance- promoting functions. Human chorionic gonadotropin, a factor secreted by the trophoblast, has already known immunological properties: It promotes the induction and activation of regulatory T cells, and these cells are necessary for the successful establishment of pregnancy^33,34^. hCG treatment of dendritic cells prior to conception induces regulatory T cell expansion and leads to decreased spontaneous abortion in mice^35^. In addition, hCG stimulates macrophage function and activation^91,92^. In our study, hCG and its downstream messenger cyclic AMP- treatments both suppress *CXCL10* expression and secretion in stromal cells. *CXCL10* suppression supports the anti-inflammatory function of hCG. *CXCL10* suppression also results in decreased CD8 + T cell migration in decidual cells compared to stromal cells. This data is in line with others who have found that hCG down-regulates CD8 + T cells in pregnancy^93^. Furthermore, hCG suppresses not only CXCL-10, but other pro-inflammatory cytokines as well, such as GRO-alpha, IL-8, IL-17, and RANTES. Kokdehoff and others also found that hCG decreases inflammatory cytokines in women treated with hCG prior to IVF^94^. However, our data go beyond mere description, and we characterize the mechanism by which the fetal factor hCG suppresses *CXCL10* expression in decidual cells as it may promote maternal tolerance.

We describe how the fetus/placental unit is an active participant in the process of fetal-maternal tolerance: It acts through hCG using epigenetic mechanisms to suppress inflammation. Our data illustrate a novel role of hCG as a functional epigenetic regulator suppressing inflammation. Treatment of stromal cells with hCG upregulates EZH2, a specific histone methyltransferase, enriching the repressive histone mark H3K27me3 in Region 4 of the *CXCL10* promoter. This enrichment results in decreased expression and secretion of CXCL-10. In addition, enrichment of the H3K27me3 (and subsequent suppression of CXCL-10) also occurred during decidualization with cyclic AMP. This data supports a role of hCG in maintaining the normal state of pregnancy.

We also show how infection can induce inflammation and break the fetal-maternal tolerance by disrupting the epigenetic mechanism. In our organ culture experiments using term uncomplicated human samples, we are able to induce *CXCL10* expression and secretion with LPS treatment. Moreover, we show that LPS treatment decreases the enrichment of H3K27me3 in Region 4 of the *CXCL10* promoter. LPS affects H3K27me3 methylation by inducing JMJD3, a histone demethylase, which is also seen in the regulation of other pro-inflammatory cytokines in HUVEC cells^95^.

In conclusion, our data demonstrates how the fetus/trophoblast unit actively participates in regulating inflammation at the maternal-fetal interface in order to promote pregnancy. The epigenetic inhibition of *CXCL10*- mediated inflammation is promoted by hCG on stromal cells. However, in cases of infection, LPS can modulate the suppression, and induce inflammatory chemokines that will recruit cytotoxic T cells to the maternal-fetal interface. Our results provide a possible mechanism by which infection can induce pregnancy complications such as preterm birth (Fig. 12).

As an anti-inflammatory agent, hCG may have therapeutic potential for prevention of miscarriage and preterm birth in pregnancies complicated by infection. Further clinical trials are needed to investigate the therapeutic role of hCG for prevention of fetal loss.

## Materials and Methods

### All methods were performed in accordance with the relevant guidelines and regulations and approved by Yale University

#### Reagents and cell line

Reagents and cell line used in the experiments were as follows: LPS isolated from E. coli 0111:B4 (Sigma-Aldrich, St. Louis, MO), GSK-J4 and GSK126 were purchased from (ApexBio, Houston, TX) and telomerase-immortalized human endometrial stromal cells (HESC) were developed in our laboratory and previously reported^52,96^.

### *In-vitro* decidualization of a uterine stromal cell line

HESC cell line was decidualized as previously described^52,96^. In short, HESC was cultured in DMEM low-glucose medium supplemented with 10% FBS (Gibco, Waltham, MA). Upon reaching confluency, the HESCs were switched to Optimem (Gibco, Waltham, MA) and treated for 7 days with 10 nM β2-estradiol, 1 μM medroxyprogesterone acetate, and 500 μM 8-bromo cyclic AMP. On day 8, the cells were trypsinized and the cell pellets were collected for RNA extraction.

### Endometrial stromal cells conditioned media preparation

Human endometrial stromal cells (HESC) were plated at 5 × 10^5^ cells/100 mm dish with 10% FBS DMEM-F12 media and allowed to attach overnight. Upon reaching confluency, the uterine stromal cells were switched to Optimem (Gibco, Waltham, MA) and differentiated into decidual cells by treating them for 5 days with 10 nM β2-estradiol, 1 μM medroxyprogesterone acetate, and 500 μM 8-bromo cyclic AMPM. Control cells received vehicle for the same length of time. After 5 days media was then changed to 1 Optimem media and incubated for additional 48 hrs. Cell supernatant was collected and spun down to remove any cellular debris. Cell free supernatant was aliquoted and stored at −80 °C until use.

### Chromatin immunoprecipitation and ChIP-PCR

Chromatin immunoprecipitation (ChIP) was performed on the treated uterine stromal cell line and the decidua tissue using the Zymo-Spin ChIP Kit according to the manufacturer’s instructions (Irvine, CA). Briefly, the tissue was mechanically homogenized, crosslinked, and then immunoprecipitation was performed using an anti-H3K27me3 antibody (Cell Signaling Technology, Danvers, MA) and a rabbit anti-IgG antibody (Santa Cruz Biotechnology, Dallas, TX). The ChIP DNA was purified and stored until they were ready for PCR.

The previously stored DNA purified after chromatin immunoprecipitation was used for PCR. Eight pairs of primers were designed to span the 1000 bp upstream of the human CXCL10gene in order to identify the binding site(s). The PCR product was then run on a 1% DNA Agarose gel and imaged using the Kodak Image Station 4000 (Carestream Health, New Haven, CT). When the location of enrichment was identified (Region 4), that primer set was used for the subsequent ChIP PCR reactions. The purified ChIP DNA was used for real-time PCR using the primers from Region 4 to quantify the enrichment of the H3K27me3 histone to Region 4 of the CXCL10 promoter.

### Real-time PCR

RNA was isolated from the samples using the Qiagen RNeasy Mini Kit (Qiagen, Waltham, MA). cDNA was prepared using the iScript cDNA Synthesis Kit (Bio-Rad, Hercules, CA) as previously described^97^.

### Luminex multiplex assay

After culture and treatment, the supernatant was collected, centrifuged, aliquoted, and stored until use. The samples were thawed only once immediately prior to running the assay. The samples were run on the Luminex Multiplex Assay (R&D Systems, Minneapolis, MN) for the following cytokines and chemokines: GROa, IL-1b, IL-6, IL-8, IL-10, IL-12, IL-17, G-CSF, GM-CSF, IFN-Y, CXCL-10, MCP-1, MIP-1a, MIP-1b, RANTES, TNF-a, and VEGF.

### Cell migration experiments

Transwell migration experiments were performed using conditioned media from the non-decidualized and decidualized uterine stromal cell line. PBMCs were seeded in 5µm-inserts (3 × 10^5^ cells/insert) (BD Falcon cell culture inserts), which then were set in a 24-well plate containing the conditioned media from the stromal cells under different treatments. After 24 hours, the cells were recovered from the lower compartment and the total number of migrating cells was quantified. In addition, the percentage of CD4 or CD8 cells were quantified by FACS analysis. The results are expressed relative to basal conditioned for each assay.

### Flow-cytometry analysis

T lymphocytes were stained with the following mAbs: mouse anti-human CD4 PE conjugated, mouse and anti-human CD8 APC conjugated, according to the manufacturer’s instructions (BD Biosciences, San Jose, CA, USA). Briefly, after recovery, the cells were washed and surface stained with anti-CD4 and anti-CD8. The levels of CD4 and CD8 T cells were then quantified.

### Western blot

Western blot was performed using a monoclonal anti- H3K27me3 antibody (Cell Signaling Technology, Danvers, MA). The cells were lysed and sonicated with cell lysis buffer (Cell Signaling Technology, Danvers, MA) supplemented with protease inhibitors. Equal amounts of protein were boiled in SDS-containing sample buffer, resolved on SDS-PAGE, and immunoblotted with the anti-H3K27me3 antibody.

### Organ culture experiments

The Institutional Review Board of Yale University approved the collection and use of these tissues for research purposes (HIC# 12696). Organ culture experiments were performed using decidual tissue isolated from the placentae of term uncomplicated non-labored pregnancies at the time of scheduled cesarean delivery. Immediately upon delivery, the decidua was separated from the fetal membranes, rinsed in phosphate buffered saline, and cut into approximately 3 × 3 mm pieces and placed in DMEM media supplemented with Normocin (InvivoGen, San Diego, CA) for 24 hours. The following day, the media was refreshed, the tissue was treated with either LPS or inhibitors, and after 24 hours, it was collected for RNA isolation or crosslinked for chromatin immunoprecipitation. Supp Fig. 1 shows a representative H&E stained slide of the decidua tissue used for the organ culture experiments.

### Chromatin immunoprecipitation

Chromatin immunoprecipitation was performed on the treated uterine stromal cell line and the decidua tissue using the Zymo-Spin ChIP Kit (Irvine, CA). Briefly, the tissue was mechanically homogenized using an Omni TH tissue homogenizer (Omni International, Kennesaw, GA) then all samples were crosslinked using 1% formaldehyde. The crosslinking reaction was stopped using 0.125 M glycine. The samples were then washed with phosphate buffered saline supplemented with protease inhibitors, centrifuged, and stored at −80 °C until ready for use. Next, the samples were sonicated to a size of approximately 250 bp size using a Sonic Dismembrator 500 (Fisher Scientific, Waltham, MA). The size of the chromatin was confirmed by running the sheared chromatin on a DNA 1% Agarose gel. After sonication, the immunoprecipitation reaction was performed using an anti-H3K27me3 antibody (Cell Signaling Technology, Danvers, MA) and a rabbit anti-IgG antibody (Santa Cruz Biotechnology, Dallas, TX) as a negative control and the ZymoMag Protein A beads supplied with the kit. The samples were washed with the provided wash buffers, then reverse crosslinked, and the resulting DNA was purified and stored until they were ready for PCR.

### ChIP-qPCR

ChIP Primers used in this study: The 8 primer sequences corresponding to the location in the CXCL10 promoter are as follows:

Region 1: F: CCATTTTCCCTCCCTAATTC, R: CCTTCGAGTCTGCAACATG

Region 2: F: GTAGCCTCCAAGTTACGG, R: GAATGGATTGCAACCTTTG

Region 3: F: CAAAGGTTGCAATCCATTC, R: CACAACTTGCTGTTACC

Region 4: F: GAACAGTGATTTACCTGGACA, R: CTTGCCAGTTCCAGATCTTTG

Region 5: F: GACAGGGTCAAAGATCTGG, R: CCCTCAAAATAGTTATGTTGG

Region 6: F: CATTGCTCATTTGGGTATCTGA, R: CAATAACCCTAGGATAGCTATG

Region 7: F: AGGGTGCTTTTTGAGGA, R: ACAGTGTCTTGGAGCTGA

Region 8: F: CTCCAAGACACTGTTAAATG, R: GCCACGATTCATCATCC

### Real-time PCR

Real-time PCR was performed using the following primers:

CXCL-10: F: CCCACGTGTTGAGATCATTG, R: TCCATCACAGCACCGGG,

JMJD3: F: AGTACCGCACTGAGGAGCTG, R: CAGTGCCCTCCTCCGCT,

EZH2: F: TGCACATCCTGACTTCTGTGA, R: TCATCTCCCATATAAGGAATGTTATG,

Prolactin: F: CATCAACAGCTGCCACACTT, R: CGTTTGGTTTGCTCCTCAAT,

IGFBP1: F: CTATGATGGCTCGAAGGCTC, R: TTCTTGTTGCAGTTTGGCAG,

GAPDH: F: TTAAAAGCAGCCCTGGTGAC, R: CTCTGCTCCTCCTGTTCGAC.

### Patient recruitment and sample collection

The description of the patients population, recruitment and characteristics has been previously described^98^ as following:

#### IVF Cohort

Recruitment of IVF patients and storage of samples was approved by the Yale institutional Review Board (IRB) with no written consent requirement (#2000021607). All the participants had an informed consent for study participation. An aliquot of blood was assigned for the study during the regular blood test for monitoring chemical pregnancy (hCG serum levels). The study was deemed to have minimal harm to patients so only verbal consent was requested. The investigators have no access to any personal information. Coded serum samples were provided to the investigators without any patient information.

Patients aged 18–44 undergoing fresh or frozen day 3 or day 5 (blastocyst) embryo transfer from October 2017 to July 2018 were eligible for participation. Exclusion criteria were patients with chronic autoimmune disease (such as lupus, thyroid antibodies, ulcerative colitis, or Crohn’s disease), diabetes and hypertension requiring medication, endometriosis confirmed by laparoscopy, or current illness (in general, we excluded patients with an underlying inflammatory process). We also excluded patients with prior pregnancy losses, unless the tissue from the loss had undergone genetic testing and was determined to be chromosomally abnormal. Patients were asked to participate at the time of embryo transfer. Blood was collected by venipuncture into 10 mL vacutainer tubes starting at the time of the first positive β-hCG, 8–12 days after embryo transfer and then every 48 hours until an intrauterine pregnancy was confirmed using transvaginal ultrasound, or when a pregnancy was deemed as biochemical based on declining β-hCG levels following an initial positive test. Samples were left at room temperature for 60 minutes to allow for clotting and then centrifuged (Thermo Scientific Sorvall ST 16, Waltham, MA) at 3,000 RPM for ten minutes at room temperature. Serum was aliquoted into 1.5 mL polypropylene RNase- and DNase-free microcentrifuge tubes and stored in −80 °C freezers until ready for testing.

### Statistical analysis

Statistical analyses was done using the Statistical Package for Social Science (SPSS) for windows and GraphPad Prism software, version 5. Differences between two groups were analyzed using Student’s t-test. The differences between multiple groups were analyzed by one-way ANOVA and Chi-square test. *P*-values less than 0.05 were considered significant. All the experiments were done in triplicate and a minimum of three independent experiments.

## Supplementary information

Supplementary information.
