# Supplementary material for: Human Chorionic Gonadotropin modulates CXCL10 Expression through Histone Methylation in human decidua
Source: Sci Rep. 2020 Apr 1;10:5785. doi: 10.1038/s41598-020-62593-9 (PMC7113245; doi:10.1038/s41598-020-62593-9)
Supplement: Supplementary file 1 — Supplementary information. [file 41598_2020_62593_MOESM1_ESM.pdf]

Human Chorionic Gonadotropin modulates CXCL10 Expression through Histone Methylation in human decidua

Michelle Silasi[1], Yuan You[1,2], Samantha Simpson[1], Janina Kaislasuo[1,3], Lubna Pal[1] , Seth Guller[1], Gang Peng[4], Rosanna Ramhorst[5], Esteban Grasso[5], Shervin Etemad[1], Sandy Durosier[1], Paulomi Aldo[1], Gil Mor[1,2]

SF1

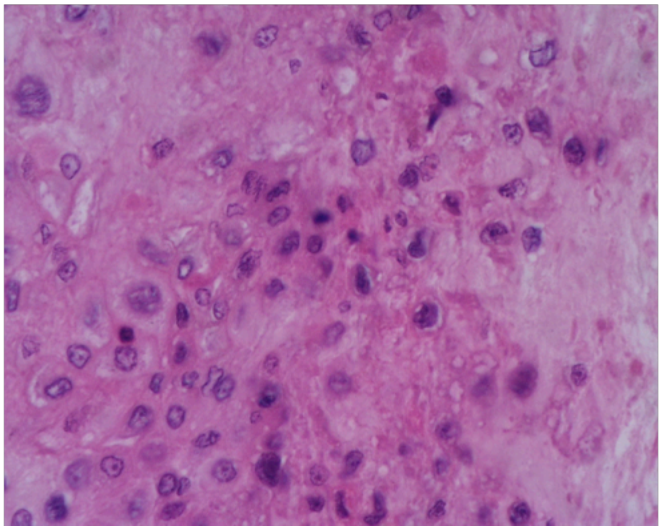

Supp Figure 1. H&E stained slide of the decidua tissue.

Representative H&E slide of decidua tissue isolated from uncomplicated term non-labored placentas taken at the time of scheduled cesarean delivery and used for organ culture experiments. 40x magnification.
